# Physical Impact of SARS-CoV-2 Infection in a Population of Italian Healthcare Workers

**DOI:** 10.3390/ijerph20054506

**Published:** 2023-03-03

**Authors:** Lucrezia Ginevra Lulli, Antonio Baldassarre, Annarita Chiarelli, Antonella Mariniello, Diana Paolini, Maddalena Grazzini, Nicola Mucci, Giulio Arcangeli

**Affiliations:** 1 Department of Experimental and Clinical Medicine, University of Florence, 50134 Florence, Italy; 2 Occupational Medicine Unit, Careggi University Hospital, 50134 Florence, Italy; 3 Occupational Medicine School, University of Florence, 50134 Florence, Italy; 4 Health Direction, Careggi University Hospital, 50134 Florence, Italy

**Keywords:** SARS-CoV-2 infection, post-COVID-19 symptoms, healthcare workers, occupational medicine, fatigue, dyspnea, musculoskeletal pain, fitness to work, BMI

## Abstract

SARS-CoV-2 infection often causes symptoms and illness that can last for months after the acute phase, i.e., so-called “Long COVID” or Post-acute COVID-19. Due to the high prevalence of SARS-CoV-2 infection among Healthcare Workers (HCWs), post-COVID-19 symptoms can be common and threaten workers’ occupational health and healthcare systems’ functioning. The aim of this cross-sectional, observational study was to present data related to post-COVID-19 outcomes in a population of HCWs infected by COVID-19 from October 2020 to April 2021, and to identify possible factors associated with the persistence of illness, such as gender, age, previous medical conditions, and features of acute illness. A total of 318 HCWs who had become infected by COVID-19 were examined and interviewed approximately two months after their recovery from the infection. The clinical examinations were performed by Occupational Physicians in accordance with a specific protocol at the Occupational Medicine Unit of a tertiary hospital in Italy. The mean age of the participants was 45 years old, and 66.7% of the workers were women while 33.3% were men; the sample mainly consisted of nurses (44.7%). During the medical examination, more than half of the workers mentioned that they had experienced multiple residual bouts of illness after the acute phase of infection. Men and women were similarly affected. The most reported symptom was fatigue (32.1%), followed by musculoskeletal pain (13.6%) and dyspnea (13.2%). In the multivariate analysis, dyspnea (*p* < 0.001) and fatigue (*p* < 0.001) during the acute stage of illness and the presence of any limitation in working activities, in the context of fitness for a work evaluation performed while the occupational medicine surveillance program was being conducted (*p* = 0.025), were independently associated with any post-COVID-19 symptoms, which were considered final outcomes. The main post-COVID-19 symptoms—dyspnea, fatigue, and musculoskeletal pain—showed significant associations with dyspnea, fatigue, and musculoskeletal pain experienced during the acute stage of infection, with the presence of limitations in working activities, and pre-existing pneumological diseases. A normal weight according to body mass index was a protective factor. The identification of vulnerable workers as those with limitations in working activities, pneumological diseases, a high BMI, and of an older age and the implementation of preventive measures are key factors for preserving Occupational Health. Fitness-to-work evaluations performed by Occupational Physicians can be considered a complex index of overall health and functionality that can identify workers who may suffer from relevant post-COVID-19 symptoms.

## 1. Introduction

Three years after the outbreak of the COVID-19 pandemic in January 2020, SARS-CoV-2 virus remains a highly diffusive pathogen that is continuing to spread globally. On 30 January 2023, according to the World Health Organization (WHO) [1], 100,000 cases were recorded within 24 h, amounting to a total number of cumulative cases of over 750 million and constituting over 6.8 million deaths from the beginning of the pandemic. In Italy, in the last week of January 2023 from 27 January to 2 February 2023, 33,042 new cases were reported by the National Health Authorities along with 439 deaths [2]. New variants of interest and concern have emerged and spread [3], such as the Omicron variant and its sublineages [4]. Massive vaccination campaigns have reduced the burden of the virus on healthcare systems worldwide, consistently protecting people against severe forms of illness [5].

COVID-19 illness is highly variable, ranging from infection with no symptoms to pneumonia and life-threatening consequences. Common symptoms are fever, cough, musculoskeletal pain, myalgia, arthralgia, headache, fatigue, and dyspnea [6]. After acute infection symptoms emerge, 10% to 20% of infected patients (and up to 45% according to a recent systematic review [7]) can experience a variety of mid- and long-term effects after they recover from their initial illness. Post COVID-19 condition, also known as “long COVID,” refers collectively to the constellation of long-term symptoms that some people experience after they have had COVID-19 [8]. The European Society of Clinical Microbiology and Infectious Disease (ESCMID) defines “Long COVID” symptoms as those persisting even 12 weeks post-infection and “Post-Acute COVID” symptoms as those persisting between 4 to 12 weeks [9]. Alternatively, according to NICE (National Institute for Health and Care Excellence, UK) guidelines, the term long COVID refers to signs and symptoms that continue after the acute stage of COVID-19 disease (4–12 weeks), and the term post-COVID-19 condition (PCC) refers to signs and symptoms that develop during or after infection with COVID-19 disease that continue for more than 12 weeks and cannot be explained by an alternative diagnosis [10,11]. In general, we can refer to the sequalae occurring after the acute stage of infection as “post-COVID”. Long COVID has been recognized as a clinical entity that may cause significant disability in not only hospitalized patients but also asymptomatic or mildly symptomatic ones [12,13,14,15]. The most commonly observed symptoms among patients with long COVID are fatigue, dyspnea, musculoskeletal pain, anosmia/dysgeusia, cognitive impairment (or brain fog), sleep disturbances, cough, and chest pain [9]. Older age, female sex, and pre-existing medical conditions (pulmonary diseases, psychiatric illness, obesity, and diabetes) have been found as possible risk factors for the development of Long COVID-19 syndrome [16,17,18]. In addition, the characteristics of the acute form of illness may have an influence on the development of post-COVID-19 symptoms [19]. The acquirement of further data is necessary to better define the clinical features of long COVID and identify strategies for its clinical management, as indicated by ESCMID and several other research authorities around the globe [20]. Omicron infection seems to present a lower prevalence of post-COVID-19 illness than previous variants, as it was prevalent in 4.5% vs. 10.8% of patients in a UK study [21], but other studies have reported higher percentages [22]. As the number of COVID-19 cases and survivors grows, the burden of post-COVID-19 illness will also increase, becoming a more relevant concern for the healthcare systems [10]. Vaccination could reduce the risk of long COVID, but further studies are needed to confirm this [23,24].

Post-COVID-19 disorders are relevant in the field of occupational medicine due to the fact that Healthcare Workers (HCWs) are continuously exposed to the risk of SARS-CoV-2 infection and its consequences. SARS-CoV-2 infection and its management by healthcare management systems remains a matter of significant concern. Omicron, the current SARS-CoV-2 variant, which includes BA.1, BA.2, BA.3, BA.4, BA.5, and descendent lineages, is much more contagious than its predecessors [25,26], which means that during surges, without an adequate level of preparedness, hospitals can become understaffed, thereby stressing and overburdening healthcare workers to a greater degree [27]. Healthcare workers are defined as workers who deliver care and services to the sick and ailing either directly (e.g., physicians, nurses, healthcare assistants, midwifes, etc.) or indirectly (e.g., aides, laboratory technicians, porters, etc.) [28]. In terms of occupational safety and health, healthcare workers are exposed to relevant biological risks, which include SARS-CoV-2 infection. In Italy, infection with SARS-CoV-2 is considered an occupational illness/accident for some categories of workers, primarily HCWs, and healthcare managers are required to adopt every necessary measure to reduce the spread of the virus to preserve workers’ and patients’ health. HCWs constitute a high-risk population that presents a 24-fold higher probability of contracting the infection than the general population [29]. HCWs’ exposure to occupational risks, including biological risks posed by SARS-CoV-2, can have different impacts on workers’ health according to their age, sex, and history of medical and psychological conditions, and occupational medicine is required to carefully evaluate such impacts to preserve occupational wellbeing. According to the literature, women and older patients may be more affected by post-COVID-19 symptoms, and some previous medical conditions can be risk factors, such as obesity and pulmonary diseases [23,30]. In a recent metanalysis analyzing 13,340 patients, female sex was associated with long COVID-19 affliction with an OR of 1.52 [31]. Post-COVID-19 sequelae can reduce functionality and work-related ability for some time, thus impacting work efficiency. This can be also a major issue in light of the highly physically and emotionally demanding tasks performed by HCWs [32]. The impact of persistent illness after being afflicted with the acute form of illness can also have negative effects on the broader healthcare delivery system, leading to a possible loss of skilled healthcare personnel due to post-COVID-19-related disabilities [33].

### Aims of the Study

The aim of this research is to provide observational data regarding post-COVID-19 outcomes in a population of HCWs infected by COVID-19 from October 2020 to March 2021. Through a cross-sectional design, herein, we present data regarding the physical health of workers two months after COVID-19 infection. We tested whether the baseline characteristics of the patients—age, sex, previous health conditions, and BMI—and the main symptoms of the acute form of the illness affected their health outcomes two months after COVID-19 infection. In addition, we hypothesized that fitness to work, which in the field of occupational medicine is the assessment of the possibility of performing a specific task, may be associated with post-COVID-19 symptoms.

## 2. Materials and Methods

Between 1 October 2020 and 30 April 2021 in a tertiary referral hospital employing approximately 5000 HCWs in Florence, Tuscany, Italy, 440 healthcare workers were infected with COVID-19 and tested positive for SARS-CoV-2 infection via a RT-PCR nasopharyngeal swab (Real-Time Polymerase Chain Reaction). After the acute phase of the illness, all the workers were contacted and invited to a post-COVID-19 return-to-work visit at the Occupational Medicine Unit. Of the 440 workers that tested positive in the considered period, 318 accepted the invitation for the medical examination, constituting a participation rate of 72.3%.

### 2.1. Definition of Healthcare Worker

We considered healthcare workers as all workers involved in the care of patients and divided them into physicians (structured doctors), nurses, healthcare assistants (workers in support of nurses and doctors, who assist patients with daily personal hygiene activities and carry out simple activities to aid nursing and technical healthcare activities), resident physicians (doctors in training), and others (radiology technicians, lab technicians, physiotherapists, midwifes, and patient transport workers).

### 2.2. Definition of COVID-19 Case

A COVID-19 case was defined via a positive RT- PCR nasopharyngeal swab, with or without the presence of COVID-19-related symptoms. The protocol of surveillance required a negative PCR swab to return to work according to the National Italian Laws for the management of the pandemic. In particular, at that time, the Health Ministry required infected individuals to undergo at-home isolation for at least 10 days after the onset of symptoms and to achieve a negative RT-PCR test at the end of the isolation period, in addition to attaining remission with respect to all COVID-19 symptoms except for alterations in smell and taste [34]. After the first positive swab, the following control swabs were programmed by the Occupational Medicine Unit after 10 days, and if they were still positive, subsequent swabs were scheduled once a week until the patient tested negative.

### 2.3. Pre-Existing Medical Conditions, Fitness to Work, and Vaccination

The patients’ pre-existing medical conditions were investigated by inquiring as to their medical histories during the medical examinations and were cross-checked with the occupational medical records available. The main pre-existing conditions uncovered were cardiovascular diseases (e.g., hypertension, cardiopathy of any kind, etc.), pneumological disease (e.g., asthma, COPD of any severity, etc.), neurological disease (e.g., epilepsy, neuropathy, etc.), psychiatric disorders (e.g., depression, anxiety requiring medical support, etc.), diabetes (type I or II), and endocrinological disorders (e.g., thyroid disorders). Diseases of any other form (e.g., breast cancer) were defined as “other disorder ”. Data regarding fitness to work, which were provided by occupational physicians, were collected retrospectively while analyzing the available medical records. The data referred to the last visit of routine medical surveillance wherein an Occupational Physician expressed the judgement for a patient’s suitability for a specific job (e.g., physician, nurse, healthcare assistant, etc.), as required by Italian national laws. A worker is judged as “completely fit to work” if his/her medical conditions allow him/her to completely perform an occupational task; thus, the clinical conditions of such a worker are completely suitable for said occupational task. Alternatively, the suitability to work can include specific limitations (e.g., the avoidance of nocturnal shifts, limitations on the amount of weight that can be handled, etc.) when required by the health status of the worker. This means that the clinical conditions of the worker allow him/her to perform the specific occupational task but with some limitations (e.g., for a healthcare assistant with back issues, a possible limitation could be that he/she cannot move patients alone; alternatively, for a nurse with decompensated type II diabetes, the limitation could be not working during night shifts). Therefore, the fitness-to-work evaluation is a comprehensive evaluation that accounts for all the clinical conditions of workers and relates them to a specific occupational task. This study took place before the rollout of the COVID-19 vaccine campaign in Italy and during the first few months when only mRNA vaccines were available. Full vaccination is defined as having received two shots of vaccine, according to the specific schedule, followed by a period of two weeks from the second dose.

### 2.4. Acute Symptoms and Post-COVID-19 Symptoms

In this study, post-COVID-19 symptoms are defined as symptoms that persist after having tested negative via RT-PCR nasopharyngeal swab and persisting at the time of a medical examination, i.e., between 4 to 8 weeks after recovery. Specific post-COVID-19 symptoms explored during the collection of data were cough, dyspnea, fatigue, headache, sleep disturbances, alteration in smell or taste, dizziness, and musculoskeletal pain. Other symptoms were defined as other forms of illness. Fatigue was defined as a state of physical asthenia that impacted typical activities to some extent. During the medical interview, workers were asked to report newly occurring or persistent symptoms after COVID-19 infection, excluding chronic issues. Workers were thus classified as suffering from any post-COVID-19 symptom when said symptom was described as having emerged during or immediately following COVID-19 infection. The information about acute and post-COVID-19 symptoms was obtained during the medical examination through an accurate recording of medical history. Notably, at the time the study was conducted, no Long/Post COVID-19 definition had officially been coined by Health Authorities or Medical Societies. Post-COVID-19 symptoms were the final outcome of the examination and concerned the presence (yes/no) of symptoms after the end of the acute stage of infection.

### 2.5. Medical Examination and Collection of Data

The medical examination occurred approximately 4 to 8 weeks following the patients’ recovery. The physicians of the Occupational Medicine Unit—who also followed the acute phase of infection through a telephonic surveillance—examined the workers and collected data about sociodemographic characteristics, previous contact with COVID-19-infected people, symptoms and length of infection, presence of post-COVID-19 symptoms, and previous medical history. In addition, a clinical examination was performed by updating the health history records, checking vital signs (e.g., blood pressure, heart rate, and respiratory rate), performing visual and physical exams, and prescribing, according to medical opinion, laboratory tests or specialist investigations. The examination was entirely carried out by specialized physicians, and the findings were reported on a specific file for each patient (see Supplementary Material for the complete form).

During the examination, a screening for post-traumatic stress disorder was performed using the IES-6 scale (Impact of Event Scale 6) [35], and questionnaires about COVID-19-related fear were administered; these psychological aspects will be addressed in a future paper. The data collection procedure was performed within a health surveillance program according to Italian Legislative Decree 81/2008. The sensitive data were collected anonymously, in accordance with the principles of the Declaration of Helsinki, and all participants gave their informed consent.

### 2.6. Statistical Analysis

IBM SPSS (Statistical Package for Social Sciences, version 29.0—IBM Corp. Armonk, NY, USA) was used to perform the statistical analysis. Univariate analysis was performed using a two-tailed chi-square test to compare the distribution of nominal data, while significative results for more than two groups were analyzed with standard residuals post hoc test. Student’s *t* test (or Mann Whitney U test if the distribution of the continuous variables was not normal) was used to compare the means between two groups.

The main outcomes were post-COVID-19 symptoms, which were analyzed as dichotomous dependent variables (e.g., fatigue: yes/no; dyspnea: yes/no; etc.). Firstly, the analysis was carried out for the whole sample; then, the sample was segregated by gender to analyze the differences between men and women. Afterwards, age was taken into account as a continuous variable, and the difference in the mean age between the two groups (presence/absence of a post-COVID-19 symptom) was analyzed through Student’s t test. Eventually, other interesting baseline variables (BMI, previous medical conditions, fitness to work, and symptoms and characteristics of the acute illness) were considered and accounted for as independent variables in the chi-square test. Correlation between continuous variables was tested with Pearson’s r or Kendall’s tau in cases of non-parametric distribution. Multiple logistic hierarchical regression including the statistically significative variables in the univariate analysis was used to perform multivariate analysis. Given the response rate, namely, 318 out of a total of 440 workers who had become infected during the study period, the sample obtained was judged to be adequate, and the significance level was set at 0.05.

## 3. Results

### 3.1. Baseline Characteristics of the Sample and the Acute Form of Infection

The data were initially analyzed as a whole sample and according to age and sex. Table S1 of the Supplementary Materials contains the baseline characteristics of the sample and their segregation according to sex and age. The participants had a mean age of 45 years old (±11.9) and comprised 212 women (66.7%) and 106 men (33.3%). The participants presented generally good health, as limitations in work activities were present in only 13% of the workers and were mainly connected to tasks involving manual labor. In addition, over two thirds of the sample were not on any medication at the time of the medical examination. The sample was primarily composed of nurses (142, 44.7%), followed by healthcare assistants (68, 21.4%), physicians (37, 11.6%), resident physicians (37, 11.6%), and others (34, 10.7%). Regarding the age distribution between the professional groups, the groups nurses and resident physicians show a lower mean age than the other groups. Generally, participants with a higher mean age show a higher burden of diseases, such as cardiovascular issues or diabetes, and take more drugs chronically. This decrease in general health through aging is also evidenced by the significative difference in the mean age between the completely fit-to-work participants and the workers with any kind of work limitation. Regarding the differences related to sex, women presented more limitations in work activities, although this is not reflected by a higher level of pre-existent medical conditions (apart from endocrinological problems). Among the male workers, there was a larger proportion of overweight workers compared to the female group, and when considering the whole sample, 11% (35) of the workers were considered obese according to their BMI (Body Mass Index). A total of 9.7% of the sample (31) was full vaccinated. The data regarding the main characteristics of the acute form of infection can be found in Table S2 of the Supplementary Material. Notably, only 10 workers were admitted to a hospital during the acute stage of illness.

### 3.2. Post-COVID-19 Symptoms

More than half of the workers (56.3%) mentioned post-COVID-19 illness, with most of these cases including more than one symptom. The most reported symptom was fatigue (32.1%), followed by musculoskeletal pain (13.6%) and dyspnea (13.2%). As determined through the univariate analysis, older workers are more likely to experience post-COVID-19 symptoms, especially in terms of musculoskeletal pain (*p* < 0.001), dyspnea (*p* = 0.030), and fatigue (*p* = 0.011). No difference was found between the male and female workers. Notably, in the acute phase of illness, there was no difference in the mean age of the group with musculoskeletal pain or fatigue and the mean age without these symptoms, while the difference in the mean age was significant when considering long-term illness. Table 1 contains the variables related to the analyzed post-COVID-19 symptoms according to sex and age.

### 3.3. Correlation between Most Common Post-COVID-19 Symptoms (Dyspnoea, Fatigue, and Musculoskeletal Pain) and Sample Characteristics

A univariate analysis was conducted to explore the relationships between the three most common post-COVID-19 symptoms (dyspnea, fatigue, and musculoskeletal pain) and the main baseline characteristics and features of the acute form of illness. The sample was divided according to the presence of any symptoms during the post-COVID-19 period and with respect to the three most common symptoms. The significative correlations are reported in Table 2. The only previous health conditions that showed a significative relationship with the post-COVID-19 symptoms were pneumological and psychiatric diseases, limitations in work activities, and BMI (considered as normal weight). Almost all symptoms and the use of medications during the acute phase of the disease show a significative relationship between the considered post-COVID-19 symptoms. The mean number of symptoms reported in the acute phase of infection was higher in the patients with the analyzed post-COVID-19 disorders. The complete results are reported in Table S3 of the Supplementary material.

### 3.4. Multivariate Analysis for Post-COVID-19 Symptoms

The variables that were found to be statistically significative in the univariate analysis were selected to be tested via multivariate analysis. Gender, as it was not found to be statistically significative, was excluded in the logistic regression. In order to analyze which variables were independently associated with post-COVID-19 disorders, four hierarchical multiple logistic regressions were performed, with each one having the following respective variables: the dependent variable, (a), the presence of any post-COVID-19 symptom; (b) post-COVID-19 dyspnea; (c) post-COVID-19 fatigue; and (d) post-COVID-19 musculoskeletal pain. Each regression consisted of two blocks. The first block included dyspnea, fatigue, and musculoskeletal pain during the acute phase of illness as predictors for (a), and age, number of acute symptoms, and the presence of dyspnea, fatigue, and musculoskeletal pain during the acute phase of illness for (b), (c), and (d), respectively. In block two, other predictors were included in the model, which were found to be statistically significative in the univariate analysis: for (a), (c), and (d), these predictors were normal weight according to BMI and limitations in work activities, while for (b), they included limitations in work activities and pre-existing pneumological diseases. The results of the logistic regression are reported in Table 3, and the details about the model and blocks tested are reported in the Supplementary Materials.

## 4. Discussion

After almost 3 years of the pandemic, COVID-19 infection and its long consequences are still relevant, especially in specific occupational contexts such as healthcare, whose workers are exposed to several occupational risks and are often overburdened. It is widely known that COVID-19 infection can result in several different ailments, denoted as post-COVID-19 symptoms or post-COVID-19 syndrome [8,36], which may last from several weeks to years [15]. These conditions challenge healthcare professionals’ return to work [37] and can transform an infection that usually lasts 1 to 3 weeks into a long, complex illness. Post-COVID-19 symptoms can affect patients with all levels of disease severity as well as young, healthy people [38,39].

This study aimed to describe the clinical post-COVID-19 outcomes of a very specific population, namely, healthcare workers who had become infected with COVID-19, to identify possible predictors of post-COVID-19 disorders. In our sample, the workers who presented several residual symptoms after having tested negative via a control swab yielded relevant results: over half of the participants mentioned at least one symptom persisting after having been declared cured and readmitted to work, which is consistent with previous works [7,30,40]. This should be also analyzed while considering the composition of our sample, that is, a working population with a mean age of 45.

The most reported disorder was fatigue, followed by dyspnea and musculoskeletal pain. This is consistent with several previous studies on COVID-19 patients’ outcomes at three months after infection [38,41,42]. For this reason, we conducted our main analysis on these three symptoms, judging them as the most relevant for the wellbeing of workers. Patients complaining of fatigue, musculoskeletal pain, and dyspnea showed a higher mean age, which is an interesting finding when considering that during the acute phase of illness, no significative difference in symptoms was reported according to either sex age. It is known that elderly patients may suffer more acutely from post-COVID-19 symptoms than younger people [43]. However, when a multivariate analysis was performed, age remained a significant factor only with respect to musculoskeletal pain. This can be explained by the fact that our sample has a relatively low average age, which may not be significative when other relevant factors are considered in the analysis.

Interestingly, no difference was found in the post-COVID-19 conditions regarding sex, and this is consistent with the findings of Petersen et al., who addressed post-COVID-19 in mild-course patients [44]. In the literature, however, female sex is considered one of the risk factors for developing post-COVID-19 disorders [45]. For example, in a review analyzing post-COVID-19 symptoms in mild-course COVID-19 patients, it was found that most common post-COVID-19 disorders occur among women (on average 60%) [46]. In a large population study of almost 400,000 patients, the risk factors for long COVID-19 included female sex [47]. In our sample, female and male workers had the same mean age, and when considering comorbidities, the two groups are very similar. This difference from the previous reported data may be related to the small size of our sample but also the different methodology with respect to the collection of data. In fact, for the most part, studies addressing post-COVID-19 disorders use self-administrated questionnaires or generic medical records. Instead, in this study, a specific post-COVID-19 medical evaluation was used to collect the data, and this could have led to the more precise reporting of symptoms, which was aided by medical personnel. In addition, this can be related to the relatively young age of our sample, which includes active, working people.

Although post-COVID-19 syndrome can also affect patients with a mild form of infection, the clinical characteristics of the acute form of infection and its severity are strictly related to the presence and severity of post-COVID-19 disorders; this relationship has already been demonstrated in previous large studies [19,36], and it was also found in our analysis. Dyspnea and fatigue during the acute phase of illness independently predicted the presence of post-COVID-19 disorders, while for each main post-COVID-19 symptom, having suffered from the same symptom during the acute phase of illness predicted its presence after recovery. For post-COVID-19 fatigue and musculoskeletal pain, the number of acute symptoms also showed a statistically significant relationship. Alternatively, for post-COVID-19 dyspnea, this relationship was not relevant. Instead, a history of pneumological diseases, such as asthma or COPD (chronic obstructive pulmonary disease), was independently related to post-COVID-19 dyspnea. In the literature, a wide range of comorbidities present at baseline assessment were associated with an increased risk of post-COVID-19 symptoms [38]; for example, associations with pre-existent medical conditions and post-COVID-19 syndrome were found for asthma and hypertension [36], and for COPD, for anxiety and depression [47]. In our sample, only post-COVID-19 dyspnea was associated with pre-existing pneumological diseases, while no other significant relationship was found with other comorbidities, which was probably due to the relatively good health of our population. Via multivariate analysis, normal weight according to BMI was found as an independent protective factor for post-COVID-19 fatigue, and this is consistent with previous literature data. High BMIs and obesity are recognized risk factors for the development of post-COVID-19 symptoms [38,47,48]. A possible explanation for this association may lie in the high levels of inflammation present in obese patients [49], which also constitutes a pathophysiological basis with which to explain the higher risk of mortality for obese patients during the acute stage of infection [50]. Remarkably, limitations in work activities constituted another factor independently linked to post-COVID-19 symptoms, particularly dyspnea and fatigue. To the best of our knowledge, this is the first study that provides a correlation between this kind of index and post-COVID-19 symptoms. According to Italian laws, occupational physicians can ascribe limitations in work activities to workers who cannot completely perform a given job for medical reasons. Therefore, fitness to work can be considered a complex index of overall health and functionality, explaining its correlation with post-COVID-19 symptoms. At the same time, post-COVID-19 symptoms can affect the fitness to work, as demonstrated by an Italian study conducted on hospitalized COVID-19-infected HCWs, where, during return-to-work examinations, fit-to-work judgements with restrictions increased from 31.4% to 58.7% [51].

### 4.1. Limitations and Strengths

This research has several limitations. This study’s cross-sectional design renders it incapable of inferring causal relations between the analyzed variables and determining a precise duration of post-COVID-19 symptoms. This study did not include controls or a longitudinal follow-up, nor an assessment of the participants’ health statuses before the infection; therefore, it serves an observational purpose and does not provide direct cause–effect relationships between COVID-19 infection and the symptoms that develop afterward. Moreover, this study did not provide a scale regarding the intensity of symptoms both during the acute illness and post-COVID-19 phases. Post-COVID-19 symptoms were explored through the recording of medical history by specialized medical personnel and based on information regarding disorders that was reported by the workers. Considering the complexity of post-COVID-19 syndrome and its associated factors, longitudinal studies are needed to explore the syndrome’s pathogenesis and clinical management. In this study, the potential bias conferred by non-respondents (those who refused to participate) should be considered; nevertheless, the response rate was acceptable. In addition, the sample was limited to a specific working population; thus, it may be not fully representative of the whole population. The relatively small sample may have affected the power of the statistical analysis. In addition, 90% of the patients were unvaccinated at the time of the study; therefore, the study does not consider the effect of widespread vaccination on post-COVID-19 outcomes. Lastly, our study relates to physical symptoms and does not investigate the psychological aspects of the disease and its sequelae, thus allowing us to reach only partial conclusions as to how a SARS-CoV-2 infection can impact health, which is defined by the WHO as psycho-physical status and well-being.

However, this study has a major point of strength: all the data were collected by physicians during a medical examination. In our opinion, this rigorous methodology adds soundness to the findings. However, this method might have led to information collection bias due to the fact that the assessment was based on workers’ reports, and some symptoms, especially fatigue, can be influenced by several external situations. In addition, as stated above, this is the first study that correlates fitness to work and post-COVID-19 symptoms.

### 4.2. Practical Implications

The experience of dealing with COVID-19 in workplaces has provided a model with which to address future infectious disease emergencies, such as surges of monkeypox or seasonal flu [52]. First of all, the identification of vulnerable groups of workers has always been a goal of occupational medicine. Our study showed that the functional evaluations conducted by occupational physicians can identify workers who may suffer from relevant post-COVID-19 symptoms. This aspect should be considered when assigning workers to certain high-risk wards, not only with respect to COVID-19 risk but, more generally, biological risk as well. BMI remains one of the modifiable risk factors for post-COVID-19 syndrome, and workplace health promotion can play an important role in reducing obesity. For example, matching an occupational examination with a clinical nutrition check-up may educate workers and, in the long term, reduce the rates of obesity among this demographic. Lastly, as acute symptoms are associated with post-COVID-19 disorders, and in mild–moderate forms as well, monitoring such patients after their recovery and return to work may be useful for addressing and managing upcoming post-COVID-19 symptoms, both from occupational and general medicine perspectives.

## 5. Conclusions

The spread of SARS-CoV-2 and its variants will continue for years [53], and, in healthcare settings, the management of the biological risk related to SARS-CoV-2 has become and will continue to be a major issue for occupational health and safety services. Post-COVID-19 symptoms, which are mostly mild to moderate, are frequent among healthcare workers infected with SARS-CoV-2, especially with respect to fatigue, dyspnea, and musculoskeletal pain, as are the relevant psychological aspects, which are not of secondary importance. Acute symptoms, BMI, and pneumological comorbidities showed significant correlations with the main post-COVID-19 symptoms, thus leading to the identification of potentially vulnerable groups of workers. Fitness-to-work evaluations, performed in the context of occupational medicine through a comprehensive and functional evaluation, were related to several post-COVID-19 symptoms, mainly dyspnea and fatigue, and may predispose high-risk workers to post-COVID symptoms after the infection.

## Figures and Tables

**Table 1:** Reported post-COVID-19 symptoms in the whole sample and segregated by sex. The table also shows the mean age according to the presence of specific post-COVID-19 symptoms.

	Total Sample	Age (Mean ± SD)	*p* Value	Men (n,%)	Women (n, %)	*p* Value
**Asymptomatic**			0.031 *			0.173
Yes	136 (42.1)	43.1 (12.3)		51 (48.1)	85 (40.1)	
No	182 (56.3)	46.2 (11.6)		55 (51.9)	127 (59.9)	
**Cough**			0.660			0.582
Yes	29 (9.1)	46 (11.6)		11 (10.4)	18 (8.5)	
No	289 (90.9)	45 (12)		95 (89.6)	194 (91.5)	
**Dyspnea**			0.030 *			0.482
Yes	42 (13.2)	48.8 (12)		12 (11.3)	30 (14.2)	
No	276 (86.8)	44.5 (11.8)		94 (88.7)	182 (85.8)	
**Fatigue**			0.011 *			0.203
Yes	102 (32.1)	47.6 (11.2)		29 (27.4)	73 (34.4)	
No	216 (67.9)	43.9 (12.1)		77 (72.6)	139 (65.6)	
**Headache**			0.859			0.123
Yes	18 (5.7)	45 (11.9)		3 (2.8)	15 (7.1)	
No	300 (94.3)	45.6 (12)		103 (97.2)	197 (92.9)	
**Other forms of illness**			0.001 **			0.119
Yes	33 (10.4)	51.5 (9.2)		7 (6.6)	26 (12.3)	
No	285 (89.6)	44.3 (12)		99 (93.4)	186 (87.7)	
**Sleep disturbances**			0.144			0.112
Yes	10 (3.1)	50.5 (10.1)		1 (0.9)	9 (4.2)	
No	308 (96.9)	44.9 (12)		105 (99.1)	203 (95.8)	
**Alteration in smell**			0.844			0.625
Yes	38 (11.9)	44.7 (11.8)		14 (13.2)	24 (11.3)	
No	280 (88.1)	45.1 (12)		92 (86.8)	188 (88.7)	
**Alteration in taste**			0.390			0.755
Yes	22 (6.9)	43 (10.4)		8 (7.6)	14 (6.6)	
No	296 (93.1)	45.2 (12)		98 (92.4)	198 (93.4)	
**Dizziness and loss of concentration**			0.947			0.158
Yes	17 (5.3)	44.9 (11.0)		3 (2.8)	14 (6.6)	
No	301 (94.7)	45.1 (12)		103 (97.2)	198 (93.4)	
**Musculoskeletal pain**			<0.001 ***			0.417
Yes	43 (13.6)	51.7 (8.9)		12 (11.3)	31 (14.6)	
No	275 (86.4)	44 (12)		94 (88.7)	181 (85.4)	

Significant results are indicated with *. * *p* < 0.05, ** *p* < 0.01, *** *p* < 0.001.

**Table 2:** Correlations between any post-COVID-19 symptom, post-COVID-19 dyspnea, post-COVID-19 fatigue, post-COVID-19 musculoskeletal pain, and baseline and acute disease characteristics of the sample.

	Any Post-COVID-19 Symptom n = 182 (n, %)	*p*	Post-COVID-19 Dyspnea n = 42 (n, %)	*p*	Post-COVID-19 Fatigue n = 102 (n, %)	*p*	Post-COVID-19 Musculoskeletal Pain n = 43 (n, %)	*p*
**Any limitation in work activities**		0.002 **		<0.001 ***				0.002 **
Yes	33 (18.1)		26 (9.5)		80 (29.3)	0.002 **	31 (11.4)	
No	149 (81.9)		16 (39)		22 (53.7)		12 (29.3)	
**Pre-existing pneumological disease**		0.381		<0.001 ***		0.041 *		0.374
Yes	17 (65.4)		11 (10.6)		13 (50)		5 (19.2)	
No	165 (56.5)		31 (42.3)		89 (30.5)		38 (13)	
**Pre-existing psychiatric disorder**		0.637		0.007 **		0.067		0.008 **
Yes	4 (66.7)		3 (50)		4 (66.7)		3 (50)	
No	178 (57.1)		39 (12.5)		98 (31.4)		40 (12.8)	
**Normal weight according to BMI**		0.007 **		0.036 *		<0.001 ***		<0.001 ***
Yes	110 (52.1)		22 (10.4)		54 (25.6)		18 (8.5)	
No	72 (67.9)		20 (18.9)		48 (45.3)		25 (23.6)	
**Any acute symptom**		<0.001 ***		0.004 **		<0.001 ***		0.003 **
Yes	174 (64.2)		42 (15.5)		100 (36.9)		43 (15.9)	
No	8 (17)		0		2 (4.3)		0	
**Use of medication during acute infection (except for NSAIDs)**		<0.001 ***		<0.001 ***		0.002 **		0.127
Yes	108 (48.4)		22 (23.4)		42 (44.7)		17 (18.1)	
No	74 (78.7)		20 (9)		60 (26.9)		26 (11.7)	
**Acute Dyspnea**		<0.001 ***		<0.001 ***		<0.001 ***		<0.001 ***
Yes	69 (88.5)		33 (42.3)		42 (53.8)		21 (26.9)	
No	113 (47.1)		9 (3.8)		60 (25)		22 (9.2)	
**Acute Fatigue**		<0.001 ***		0.030 *		<0.001 ***		<0.001 ***
Yes	134 (75.3)		30 (16.9)		87 (48.9)		35 (19.7)	
No	48 (34.3)		12 (8.6)		15 (10.7)		8 (5.7)	
**Acute musculoskeletal pain**		<0.001 ***		0.019 *		<0.001 ***		<0.001 ***
Yes	118 (71.1)		29 (17.5)		72 (43.4)		40 (24.1)	
No	64 (42.1)		13 (8.5)		30 (19.7)		3 (2)	
**Number of acute symptoms (mean±sd)**	5.2 (2.5)	<0.001 ***	6.2 (2.4)	<0.001 ***	5.7 (2.3)	<0.001 ***	6.3 (2.4)	<0.001 ***

Significant results are indicated with *. * *p* < 0.05, ** *p* < 0.01, *** *p* < 0.001.

**Table 3:** Summary of results for the final multiple logistic regression models.

	Any Post-COVID-19 Symptom		Post-COVID-19 Dyspnea		Post-COVID-19 Fatigue		Post-COVID-19 Musculoskeletal Pain	
	Exp (B), 95% CI	*p*	Exp (B), 95% CI	*p*	Exp (B), 95% CI	*p*	Exp (B), 95% CI	*p*
Age	1.0 [0.99–1.03]	0.519	1 [0.963–1.04]	0.995	1.01 [0.99–1.04]	0.271	1.059 [1.02–1.1]	0.004 **
Number of acute symptoms	--	--	1.09 [0.904–1.32]	0.434	1.24 [1.09–1.41]	0.001 **	1.22 [1.03–1.45]	0.022 *
Dyspnea	5.16 [2.4–11.3]	<0.001 ***	13.89 [4.89–39.47]	<0.001 ***	--	--	--	--
Fatigue	3.86 [2.2–6.8]	<0.001 ***	--	--	4.00 [1.91–8.38]	<0.001 ***	--	--
Musculoskeletal pain	1.59 [0.91–2.78]	0.107	--	--	--	--	8.3 [2.30–30.89]	0.002 **
Normal weight according to BMI	0.63 [0.36–1.12]	0.119	--	--	0.47 [0.27–0.84]	0.010 **	0.49 [0.23–1.03]	0.059
Limitation in work activities	2.9 [1–7.3]	0.025 *	4.79 [1.70–13.49]	0.003 **	2.31 [1.01–5.28]	0.048 *	1.67 [0.66–4.22]	0.277
Pneumological disease	--	--	4.9 [1.53–15.77]	0.008 **	--	--	--	--

Significant results are indicated with *. * *p* < 0.05, ** *p* < 0.01, and *** *p* < 0.001.

## Data Availability

Data supporting the reported results are available on request.

## Supplementary Materials

The following supporting information can be downloaded at: https://www.mdpi.com/article/10.3390/ijerph20054506/s1, File S1: Supplementary Tables; Table S1: Baseline characteristics of the sample according to sex and age. Table S2: Main clinical characteristics of the acute phase of infection for the whole sample and according to sex and age. Table S3: Associations between any post-COVID-19 symptom, post-COVID-19 dyspnea, post-COVID-19 fatigue, post-COVID-19 musculoskeletal pain, and baseline and acute disease characteristics of the sample. Paragraph S4: Multiple Logistic regression. File S2: Post-COVID-19 form.
