# Supplementary material for: Physical Impact of SARS-CoV-2 Infection in a Population of Italian Healthcare Workers
Source: Int J Environ Res Public Health. 2023 Mar 3;20(5):4506. doi: 10.3390/ijerph20054506 (PMC10002388; doi:10.3390/ijerph20054506)
Supplement: Supplementary file 1 [file ijerph-20-04506-s001.zip › Supplementary Material file S1.pdf]

Supplementary Material Tables

Table S1. Baseline characteristics of the sample and according to sex and age.

	**Total sample (n,%)**	**Age (mean ±sd)**	**P value**	**Male (n,%)**	**Female (n, %)**	**P value**
**Age (mean ±sd)**	45,1 (±11,9)	--	--	--	--	--
Sex			0.108			--
Male	106 (32,8),	43,5 (12,6)		--	--	
Female	212 (66,7)	45,8 (11,5)		--	--	
**Completely fit to work**			<.001***			0.047*
Yes	273 (85,8)	43,8 (7,5)		96 (92,3)	177 (84,3)	
Limitations in work activities	41 (13,1)	54,3 (11.8)		8 (7,7)	33 (15,7)	
**Manual handling limitations**			<.001***			0.002**
Yes	29 (9,2)	53,1 (7,7)		2 (1,2)	27 (12,9)	
No	285 (90,8)	44,4 (12)		102 (98,1)	183 (87,1)	
**Night shifts limitations**			0.002**			0.676
Yes	13 (4,1)	55,3 (6,7)		5 (4,8)	8 (3,8)	
No	301 (95,9)	44,7 (11,9)		99 (95,2)	202 (96,2)	
**Type of job**			<.001***			<.001***
Nurse	142 (44,7)	44,0 (11,1)	N-HC.Ass***, N-Oth***, N-MD*, N-Res.MD***, HC.Ass-Res.MD***, Oth.-Res.MD***,MD-Res.MD***	39 (27,5)	103 (72,5) †	
Healthcare assistants	68 (21,4)	49,8 (9,0)		15 (22,1)	53 (77,9) †	
Other	34 (10,7)	52,6 (9,7)		12 (35,3)	22 (64,7)	
Physician	37 (11,6)	49,2 (12,4)		21 (56,7)	16 (43,2)) †	
Resident physicians	37 (11,6)	29,4 (2,4)		19 (51,4)	18 (48,6) †	
**Frontline workers (COVID ward, emergency department)**			0.03*			0.323
Yes	37 (11,6)	41,4 (10,3)		15 (14,2)	22 (10,4)	
No	281 (88,4)	45,6 (12,1)		91 (85,8)	190 (89,6)	
**Pre-existing cardiovascular disease**			<.001***			0.088
Yes	45 (14,2)	55,3 (8,9)		20 (18,9)	25 (11,8)	
No	273 (85,8),	43,4 (11,5)		86 (81,1)	187 (88,2)	
**Pre-existing pneumological disease**			0.691			0.111
Yes	26 (8,2)	46 (12,5)		5 (4,7)	21(9,9)	
No	292 (91,8)	45 (11,9)		101 (95,3)	191 (90,1)	
**Pre-existing neurological disease**			0.564			0.151
Yes	9 (2,8)	43,3 (9,7)		1 (0,9)	8 (3,8)	
No	309 (97,2)	45 (12)		105 (99)	204 (96,2)	

**Pre-existing psychiatric disorder**			0.207			0.382
Yes	6 (1,9)	51,2 (7,5)		1 (0,9)	5 (2,4)	
No	312 (98,1)	44,9 (12)		105 (99)	207 (97,6)	
**Diabetes**			<.001***			0.317
Yes	yes 13 (4,1)	58,2 (4,3)		6 (5,7)	7 (3,3)	
No	305 (95,9)	44,5 (11,8)		100 (94,3)	205 (96,7)	
**Endocrinological disorder**			<.001***			0.001**
Yes	30 (9,4)	53,5 (8,8)		2 (1,9)	28 (13,2)	
No	288 (90,6)	44,2 (12)		104 (98,1)	184 (86,8)	
**Other health issues**			<.001***			0.169
Yes	65 (20,4)	50,3 (11)		17 (16)	48 (22,6)	
No	253 (79,6)	43,7 (11,8)		89 (84)	164 (77,4)	
**Number of drugs taken daily**			<.001***			0.119
0 drugs	202 (63,7)	42,3 (11,3)	0-≤2***, 0-≥3***, ≤2-≥3**	74 (69,8)	128 (60,7)	
≤2 drugs	89 (28,1)	48 (11,7)		22 (20,8)	67 (31,8)	
≥3 drugs	26 (8,2)	55,9 (9,2)		10 (9,4)	16 (7,6)	
**BMI**			<.001***			0.003**
Underweight	7 (2,2)	37,7 (10,3)	Under-obesity*, normal-over*, normal-obesity*	0	7 (3,3)	
Normal weight	210 (66,3)	43,5 (11,9)		62 (58,5)	148 (70,1) †	
Overweight	65 (20,5)	48,1 (10,7)		33 (31,1)	32 (15,2) †	
Obesity	35 (11,0)	50,3 (12,3)		11 (10,4)	24 (11,4)	
**Previous full vaccination**			0.964			0.082
Yes	31 (9,7)	45,2 (10,9)		6 (5,7)	25 (11,8)	
No	287 (90,3)	45,1 (12,1)		100 (94,3)	187 (88,2)	

*Significant results are indicated with *. *p<0.05, ** p < 0.01, *** p < 0.001; † Significant difference between observed and expected frequency at post hoc analysis.

Table S2. Main clinical characteristics of the acute infection for the whole sample and according to sex and age. Analysing the characteristics of acute infection there was no difference in the mean age for acute, except for dyspnoea (p=0.012) and diarrhoea (p=0.014) who ap-peared in patients with a higher mean age. Mean age in hospitalized patients and who used medications, except for NSAIDs, was higher (respectively p=0.020 and p<.001) too. This may be due to a more severe illness that required use of specific drugs. Non relevant differences were found between male and females for the clinical characteris-tics of the acute illness, apart from dyspnoea which occurred more often in women (0.012). Also, female workers tended to report more other disturbs (p<.001).

**Acute infection features**						
	**Total sample**	**Age (mean ±sd)**	**P value**	**Male (n,%)**	**Female (n, %)**	**P value**
**Antibiotics**			**<.001*****			0.899
Yes	49 (15,5)	50,9 (11,3)		16 (15,1)	33 (15,6)	
No	268 (84,5)	44 (11,8)		90 (84,9)	178 (84,4)	
**Antipyretics/NSAIDs**			0.929			0.203
Yes	231 (72,9)	45,1 (11,7)		24 (22,6)	62 (29,4)	
No	86 (27,1)	45 (12,1)		82 (77,4)	149 (70,6)	
**Corticosteroids**			**<.001*****			0.868

Yes	73 (23,0)	49,7 (11,1)		25 (23,6)	48 (22,7)	
No	244 (77,0)	43,7 (11,9)		81 (76,4)	163 (77,3)	
**LMW heparin**			0.090			0.492
Yes	42 (13,2)	48 (11,3)		16 (15,1)	26 (12,3)	
No	275 (86,8)	44,6 (12)		90 (84,9)	185 (87,7)	
**Use of medication (except for NSAIDs)**			**<.001*** **			0.709
Yes		48,7 (11,4)		30 (28,3)	64 (30,3)	
No		43,5 (11,9)		76 (71,7)	147 (69,7)	
**Hospitalization**			**0.020***			0.069
Yes	10 (3,1)	53,7 (10,8)		6 (5,7)	4 (1,9)	
No	308 (96,9)	44,8 (11,9)		100 (94,3)	208 (98,1)	
**Other symptoms**			0.215			**<.001*** **
Yes	65 (20,4)	46,7 (11,9)		10 (9,4)	55 (25,9)	
No	253 (79,6)	44,6 (11,9)		96 (90,6)	157 (74,1)	
**Fever**			0.653			0.340
Yes	147 (46,2)	45,4 (11,5)		53 (50)	94 (44,3)	
No	171 (53,8)	44,8 (12,3)		53 (50)	118 (55,7)	
**Cough**			0.537			0.682
Yes	119 (37,4)	45,6 (12)		38 (35,8)	81 (38,2)	
No	199 (62,6)	44,7 (11,9)		68 (64,2)	131 (61,8)	
**Dyspnea**			**0.012***			**0.027***
Yes	78 (24,5)	48 (11,8)		18 (17)	60 (28,3)	
No	240 (75,5)	44,1 (11,8)		88 (83)	152 (71,7)	
**Fatigue**			0.504			0.129
Yes	178 (56)	45,5 (12)		53 (50)	125 (59)	
No	140 (44)	44,6 (11,9)		53 (50)	87 (41)	
**Headache**			0.675			0.367
Yes	119 (37,4)	44,7 (11,7)		36 (34)	83 (39,2)	
No	199 (62,6)	45,3 (12,1)		70 (66)	129 (60,8)	
**Conjunctivitis**			0.504			0.247
Yes	26 (8,2)	47 (12,1)		6 (5,7)	20 (9,4)	
No	292 (91,8)	44,9 (12,1)		100 (94,3)	192 (90,6)	
**Musculoskeletal pain**			0.869			0.578
Yes	166 (52,2)	45,2 (11,7)		53 (50)	113 (53,3)	
No	152 (47,8)	45 (12,2)		53 (50)	99 (46,7)	
**Alteration in smell**			0.871			0.874
Yes	148 (46,4)	45 (12)		50 (47,2)	98 (46,2)	
No	170 (53,6)	45,2 (12)		56 (52,8)	114 (53,8)	
**Alteration in taste**			0.985			0.420
Yes	130 (40,9)	45,1 (11,8)		40 (37,7)	90 (42,5)	
No	188 (59,1)	45,1 (12,1)		66 (62,3)	122 (57,5)	
**Dizziness**			0.274			0.414
Yes	20 (6,3)	47,9 (10,6)		5 (4,7)	15 (7,1)	
No	298 (93,7)	44,9 (12)		101 (95,3)	197 (92,9)	
**Nausea and vomit**			0.194			0.061
Yes	12 (3,8)	49,8 (8)		1 (0,9)	11 (5,2)	
No	306 (96,2)	44,9 (12)		105 (99,1)	201 (94,8)	
**Diarrhea**			**0.014***			0.765
Yes	63 (19,8)	48,4 (11,2)		20 (18,9)	43 (20,3)	
No	255 (80,2)	44,3 (12)		86 (81,1)	169 (79,7)	
**Asymptomatic**			0.265			0.146
Yes	47 (14,8)	43,3 (12,7)		20 (18,9)	27 (12,7)	
No	271 (85,2)	45,4 (11,8)		86 (81,1)	185 (87,3)	

Length of COVID infection (swab positivity)			0.457			0.570
<14 days	42 (13,2)	42,9 (11,4)		12 (11,3)	30 (14,1)	
14-21 days	60 (18,9)	45,6 (11,7)		23 (21,7)	37 (17,5)	
>21 days	216 (67,9)	45,3 (12,1)		71 (67)	145 (68,4)	
	**Total sample**	**Kendall's tau b**	**P value**	**Male (mean ±sd)**	**Female (mean ±sd)**	**P value**
**Number of acute symptoms**	4 (2,8)	0.057 (Kendall)	0.151	3,6 (2,7)	4,2 (2,8)	0.082

*Significant results are indicated with *. *p<0.05, ** p < 0.01, *** p < 0.001.*

Table S3. Associations between any post-COVID symptom, post-COVID dyspnoea, post-COVID fatigue, post-COVID musculoskeletal pain and baseline and acute disease's characteristics of the sample.

	**Any post-COVID symptom (n=182)**	**p**	**Post-COVID dyspnea (n=42)**	**p**	**Post-COVID Fatigue (n=102)**	**p**	**Post-COVID Musculoskeletal Pain (n=43)**	**p**
**Pre-existing cardiovascular disease**		0.686		0.146		0.881		0.368
Yes	27 (60)		9 (20)		14 (31,1)		8 (17,8)	
No	155 (56,8)		33 (12,1)		88 (32,2)		35 (12,8)	
**Pre-existing neurological disease**		0.431		0.418		0.521		0.229
Yes	4 (44,4)		2 (22,2)		2 (22,2)		0 (0)	
No	178 (57,6)		40 (12,9)		100 (32,3)		43 (13,9)	
**Diabetes**		0.372		0.056		0,267		0.841
Yes	9 (69,2)		4 (30,8)		6 (46,2)		2 (15,4)	
No	173 (56,7)		38 (12,5)		96 (31,5)		41 (13,4)	
**Endocrinological disorder**		0.478		0.557		0.329		0.597
Yes	19 (63,3)		5 (16,7)		12 (40)		5 (16,7)	
No	163 (56,6)		37 (12,8)		90 (31,3)		38 (13,2)	
**Previous full vaccination**		0.153		0.084		0.702		0.510

Yes	14 (45,2)		1 (3,2)		9 (29)		3 (9,7)	
No	168 (58,5)		41 (14,3)		93 (32,4)		40 (13,9)	
**Length of COVID infection (swab positivity)**		0.221		0,150		0,035		0,412
<14 days	19 (45)		3 (7,1)		11 (26,2)		3 (7,1)	
14-21 days	34 (56,7)		5 (8,3)		12 (20)		8 (13,3)	
>21 days	129 (59,7)		34 (15,7)		79 (36,6)		32 (14,8)	
**Hospitalization**		0.033*		<,001***		<,001***		0,013*
Yes	9 (90)		7 (70)		8 (80)		4 (40)	
No	173 (56,2)		35 (11,4)		94 (30,5)		39 (12,7)	
**Fever**		<.001***		0.012*		<.001***		0.003**
Yes	100 (68,3)		27 (18,4)		67 (45,6)		29 (19,7)	
No	82 (48)		15 (8,8)		35 (20,5)		14 (8,2)	
**Cough**		<.001***		0.001**		<.001***		0.324
Yes	91 (76,5)		25 (21)		54(45,4)		19 (16)	
No	91 (45,3)		17 (8,5)		48 (24,1)		24 (12,1)	
**Headache**		<.001***		0.005**		<.001***		<.001***
Yes	86 (72,3)		24 (20,2)		58 (48,7)		27 (22,7)	
No	96 (48,2)		18 (9)		44 (22,1)		16 (8)	
**Alteration in smell**		<.001***		0.013*		0.005**		0.049*
Yes	108 (73)		27 (18,2)		59 (40)		26 (17,6)	
No	74 (43,5)		15 (8,8)		43 (25,3)		17 (10)	
**Alteration in taste**		<.001***		0.008**		0.003**		0.032*
Yes	95 (73,1)		25 (19,2)		54 (41,5)		24 (18,5)	
No	87 (46,2)		17 (9)		48 (25,5)		19 (10,1)	
**Dizziness**		0.097		0.022*		0.006**		0.004**
Yes	15 (75)		6 (30)		12 (60)		7 (35)	
No	167 (56)		36 (12,1)		90 (30,2)		36 (12,1)	

**Nausea and vomit**		0.014*		0.718		0.047*		0.041*
Yes	11 (91,7)		2 (16,7)		7 (58,3)		4 (33,3)	
No	171 (55,9)		40 (13,1)		95 (31)		39 (12,7)	
**Diarrhea**		<.001***		0.266		<.001***		0.002**
Yes	50 (79,4)		11 (17,5)		32 (50,8)		16 (25,4)	
No	132 (51,8)		31 (12,2)		70 (27,5)		27 (10,6)	
**Other symptoms**		<.001***		0.002**		0.348		0.034*
Yes	50 (76,9)		16 (24,6)		24 (36,9)		14 (21,5)	
No	132 (52,2)		26 (10,3)		78 (30,8)		29 (11,5)	

Paragraph S4

Multiple Logistic regression

3.3.1. a) Any post-COVID symptom

The results showed that the first model including age, the presence of dyspnoea, fatigue and musculoskeletal pain in the acute phase of infection was significant (χ^2 (4)=84,3, p<0.001), with a Nagelkerke R-squared of .318 and a non-significant Hosmer-Lemeshow test (p=0,236). In this model dyspnoea (Exp(B)=5,48, 95% CI=2,5-11,9, p<.001) and fatigue (Exp(B)=3,73, 95% CI=2,16-6,47, p<.001) in the acute illness were statistically associated with the presence of any post-COVID symptom. The second model showed significant improvement from the first block (χ^2 (6)=91,52, p<0.001), with a Nagelkerke R-squared of .341 and a non-significant Hosmer-Lemeshow test (χ^2(8)=5,1, p=0.747). Dyspnoea (Exp(B)=5,16, 95%CI=2,4-11,3, p<.001), fatigue (Exp(B)=3,86, 95% CI=2,2-6,8, p<.001) and any limitation in work activities (Expo(B)=2,9, 95%CI=1.1-7.3, p=0.025) were independently associated with the presence of any post-COVID symptom

3.3.2. b) Dyspnoea

The results showed that the first model including age, number of symptoms and dyspnoea during the acute infection significant (χ^2 (3)=67,20, p<0.001), with a Nagelkerke R-squared of .354 and a non-significant Hosmer-Lemeshow test (p=0,252). In this model dyspnoea (p<.001, Exp(B)=12,3, 95% CI=4,7-31,8) in the acute illness was statistically associated with the presence of post-COVID dyspnoea. The second model showed significant improvement from the first block (χ^2 (5)=86,47, p<0.001), with a Nagelkerke R-squared of .442 and a non-significant Hosmer-Lemeshow test (χ^2(8)=1,925, p=0.983). Dyspnoea (Exp(B)=13,9, 95% CI=4,9-39,5, p<.001), pre-existing pneumological diseases (Exp(B)=4,9, 95% CI=1,5-15,8 p<.008) and any limitation in work activities (Exp(B)=4,8, 95% CI=1,7-13,5, p=0.003) were independently associated with the presence of post-COVID dyspnoea.

3.3.3. Fatigue

The results showed that the first model including age, number of symptoms and fatigue during the acute infection significant (χ^2 (3)=74,9, p<0.001), with a Nagelkerke R-squared of .297 and a non-significant Hosmer-Lemeshow test (p=0,802). In this model age (Exp(B)=1,026, 95%CI=1-1,05, p=0.025), fatigue in the acute illness (Exp(B)=3,6, 95%CI=1,7-7,4, p<.001) and the number of acute symptoms (Exp(B)=1,27,

95% CI=1,11-1,4, p<0.001) were statistically associated with the presence of post-COVID fatigue. The second model showed significant improvement from the first block (χ^2 (5)=84,6, p<0.001), with a Nagelkerke R-squared of .330 and a non-significant Hosmer-Lemeshow test (χ^2(8)=10,01,p=0.265). Fatigue (Exp(B)=4,0, 95% CI=1,9-8,4, p<.001), number of acute symptoms (Exp(B)=1,23, 95% CI=1,1-1,4, p=0.001), any limitation in work activities (Exp(B)=2,3, 95% CI=1,01-5,28, p=0.048) were independently associated with the presence of post-COVID fatigue, while being normal weighted at BMI was a protective factor (Exp(B)=0,472, 95% CI=0.27-0.84p=0.010). Age was no longer an independent predictor (p=0,27).

3.3.4. Musculoskeletal pain

The results showed that the first model including age, number of symptoms and fatigue during the acute infection significant (χ^2 (3)=63,3, p<0.001), with a Nagelkerke R-squared of .332 and a non-significant Hosmer-Lemeshow test (p=0,492). In this model age (Exp(B)=1,1, 95% CI=1,03-1,11, p<.001), musculoskeletal pain in the acute illness (Exp(B)=9,01, 95% CI=2,44-33,3, p<.001) and the number of acute symptoms (Exp(B)=1,25, , 95% CI=1,05-1,47, p=0.010) were statistically associated with the presence of post-COVID musculoskeletal disturbs. The second model showed significant improvement from the first block (χ^2 (5)=67,7, p<0.001), with a Nagelkerke R-squared of .353 and a non-significant Hosmer-Lemeshow test (χ^2(8)=9,76 p=0.282). Age (Exp(B)=1,06, 95% CI=1,02-1,1, p=0.004), musculoskeletal pain (Expo(B)=8,3, 95%CI=2,23-30,89, p=0.002, OR) and the number of acute symptoms (Exp(B)=1,22, 95% CI=1,03-1,45, p=0.022) remained independently associated with the presence of musculoskeletal disorders during the post-COVID phase, while having a normal BMI and limitations in work activities did not reach the level of significance.
