# Supplementary material for: Physical Impact of SARS-CoV-2 Infection in a Population of Italian Healthcare Workers
Source: Int J Environ Res Public Health. 2023 Mar 3;20(5):4506. doi: 10.3390/ijerph20054506 (PMC10002388; doi:10.3390/ijerph20054506)
Supplement: Supplementary file 1 [file ijerph-20-04506-s001.zip › Supplementary Material file S2 POST COVID FORM.pdf]

# POST-CoViD-19 RETURN



| PERSONAL DATA AND EMPLOYMENT CARD | | | |
|---|---|---|---|
| SURNAME | | FIRST NAME | |
| REGISTER | | TYPE | |
| MOBILE PHONE | | E-MAIL | |
| TASK | | DEPARTMENT | |

| SARS-CoV-2 RT-PCR | | |
|---|---|---|
| | **DATE** | **RESULT** |
| **RT-PCR** | | |
| **RT-PCR** | | |
| **RT-PCR** | | |
| **RT-PCR** | | |

| ACTIVE SURVEILLANCE | |
|---|---|
| **CLASSIFIED** | |
| **INDEX FROM RISK MdL (self classified)** | |
| **CONTACTS OUTSIDE WORK** | |

| CoViD-19 COURSE | | | |
|---|---|---|---|
| **HOSPITALIZATION** | | | |
| **FEVER** | | **ANOSMY** | |
| **COUGH** | | **AGEUSIA** | |
| **DYSPNEA** | | **CONFUSION** | |
| **ASTHENIA** | | **VOMIT** | |
| **headache** | | **DIARRHEA** | |
| **CONJUNCTIVITIS** | | **ASYMPTOMATIC** | |
| **PAINS OSTEO-MUSCLE-ARTICLES** | | **OTHER** | |
| **DURATION POSITIVITY** | | | |
| **NOTE / THERAPY** | | | |

| POST CoViD-19 | | | |
|---|---|---|---|
| **ASYMPTOMATIC** | | **ANOSMY** | |
| **COUGH** | | **AGEUSIA** | |
| **DYSPNEA** | | **CONFUSION** | |
| **ASTHENIA** | | **CONJUNCTIVITIS** | |
| **headache** | | **OSTEUM PAIN-MUSCLE-JOINTS** | |
| **NOTE / THERAPY** | | | |

# POST-CoViD-19 RETURN



| REMOTE PATHOLOGICAL HISTORY | | |
|---|---|---|
| m. CARDIOVASCULAR | | |
| m. PNEUMOLOGICAL | | |
| m. NEUROLOGICAL | | |
| m. PSYCHIATRIC | | |
| DIABETES | | |
| OTHER | | |
| FRAGILITY | | |

| PHARMACOLOGICAL HISTORY | |
|---|---|
| CHRONIC HERAPIES IN ACT | |
| NOTE | |

| OBJECTIVE EXAMINATION | | | |
|---|---|---|---|
| WEIGHT | | HEIGHT | |
| PA ( mmHg ) | | FC ( bpm ) | |
| CARDIOVASCULAR | | | |
| PNEUMOLOGICAL | | | |
| NEUROLOGICAL | | | |
| PSYCHIATRIC | | | |
| OTHER | | | |

| FOLLOW UP | | | | |
|---|---|---|---|---|
| | DATE | IgG | Value | IgM |
| SEROLOGY | | | | |
| SEROLOGY | | | | |
| SEROLOGY | | | | |
| TAKEN IN CHARGE | ☐ CARDIOLOGICAL | ☐ PNEUMOLOGICAL | ☐ NEUROLOGICAL | ☐ PSYCHOLOGICAL |
| OTHER | | | | |

Medical Specialist in Training ☐ Occupational Medical Director

_______________________ _______________________

| IES-6 Stairs | |
|---|---|
| 1. Since the beginning of the emergency, many things have made me think about COVID-19 | |
| 2. Since the beginning of the COVID-19 emergency, I have been thinking about it even without wanting to | |
| 3. Since the beginning of the COVID-19 emergency, I have been feeling nervous and alarmed | |
| 4. Since the beginning of the COVID-19 emergency, I try not to think about it | |
| 5. Since the beginning of the COVID-19 emergency, I realize that I still have many negative emotions without realizing it | |
| 6. Since the start of the COVID-19 emergency, I have had difficulty concentrating | |

| How scared he was from the risk of | |
|---|---|
| 1. Getting infected with SARS-CoV-2 / CoViD-19 | |
| 2. Being quarantined | |
| 3. Aggravate existing diseases | |
| 4. Infecting family members | |

| | | | | | | | | | |
|---|---|---|---|---|---|---|---|---|---|
| 1 | For Nothing | 2 | In Part | 3 | Enough | 4 | In Good Part | 5 | Extremely |
